# Brain abscess and heart: the phantom menace?

**DOI:** 10.1111/ene.16315

**Published:** 2024-04-23

**Authors:** Pacôme Constant dit Beaufils, Julien Plessis, Benoît Guillon

**Affiliations:** ^1^ CHU Nantes, Stroke Unit, CNRS, INSERM, l'institut du thorax Nantes Université Nantes France; ^2^ CHU Nantes, Cardiology Department, l'institut du thorax Nantes Université Nantes France; ^3^ CHU Nantes, Stroke Unit Nantes Université Nantes France

As neurovascular practitioners, we were intrigued by Bodilsen et al.'s study [1]. Our colleagues from infectious disease or neurosurgery departments recurrently seek our insights on the link between patent foramen ovale (PFO) and brain abscesses, and the potential benefits of PFO closure.

Bodilsen et al. investigate right‐to‐left shunt (RLS) prevalence using agitated saline contrast echocardiography in cryptogenic brain abscess patients. The abscess aetiology and diagnostic procedures to determine the underlying cause remain unresolved. Yet, their findings suggest that occult pulmonary arteriovenous malformations (PAVMs) are not a prevalent predisposing factor in most cases [1].

Approximately 20% of brain abscesses are cryptogenic, with the predominant bacteria often indicating an origin in the oral cavity [1]. Interestingly, this bacterial flora closely resembles that found in hereditary haemorrhagic telangiectasia‐associated abscesses, supporting the hypothesis of systemic haematogenous spread from the oral cavity [1].

Pulmonary arteriovenous malformations are not the predominant cause of RLS, unlike PFO, which is the most common congenital cardiac anomaly, affecting up to 25% of the adult population. PFO often results in varying degrees of RLS. Initially identified as a risk factor for acute ischaemic stroke (AIS) in adults under 55, with a two to three times higher risk compared to the general population, PFO is now recognized as a risk factor for AIS recurrence and eventually as a definitive aetiology. Consequently, AIS cases in adults under 60 with a normal aetiological evaluation except for the presence of a PFO are no longer classified as cryptogenic but are attributed to cardioembolic origins, warranting closure to mitigate the risk of recurrence [2].

Bodilsen et al. used Barzilai et al.'s methodology [3], distinguishing between PAVM and PFO patterns. Specifically, PAVM patterns manifest as bubbles within the left‐sided cardiac chambers after the initial three cardiac cycles, whereas PFO patterns emerge within the first three cardiac cycles. Amongst 45 cases, 10 (22%) showed PAVM patterns and nine (20%) showed PFO patterns; only one patient had confirmed PAVM through chest computed tomography. These undetermined sources of RLS may be attributed to PFO, rather than micro PAVMs. Indeed, an RLS occurring after three cardiac cycles may also be associated with a PFO, as variations in cardiac anatomy can impact the passage of bubbles. According to this assumption, in this study, intracardiac RLS prevalence appears to be greater than in a healthy population (45%) [2]. Moreover, provocative manoeuvres during echocardiography, particularly transthoracic echocardiography, known for its superior comfort compared to transoesophageal echocardiography, were not conducted, despite their potential to increase PFO detection sensibility by up to 50% [4].

In conclusion, we concur with Bodilsen et al. on PAVM's rarity as a cause of cryptogenic brain abscess. However, drawing from our understanding of PFO and cryptogenic AIS, we propose that PFO could account for a portion of cryptogenic abscess cases, perhaps through RLS and accompanying bacteraemia or septic paradoxical embolism.

Further studies should investigate systemic haematogenous bacteraemia, possibly involving PFO, using methods akin to Bodilsen et al.'s with provocative manoeuvres or exploring alternative spreading pathways through direct venous or lymphatic drainage.

## AUTHOR CONTRIBUTIONS

Conceptualization; investigation; writing—original draft: Pacôme Constant dit Beaufils. Conceptualization; validation; writing—review and editing: Julien Plessis. Conceptualization; writing—original draft; writing—review and editing: Benoît Guillon.

## CONFLICT OF INTEREST STATEMENT

All authors declare no conflict of interest.

## Data Availability

Data sharing not applicable to this article as no datasets were generated or analysed during the current study.
